# Reduced Recovery of Depression in Female T Allele Carriers of *TNF-RII* rs1061622 at Earlier Stage after Wenchuan Earthquake

**DOI:** 10.3390/ijerph15061075

**Published:** 2018-05-25

**Authors:** Nazakat Hussain Memon, Mei Fan, Jia Lin, Yan Jun Si, Mi Su, Qi Wei Guo, Ding Zhi Fang

**Affiliations:** Department of Biochemistry and Molecular Biology, West China School of Basic Medical Sciences and Forensic Medicine, Sichuan University, Chengdu 610041, China; nazakat31@hotmail.com (N.H.M.); fan_mei@163.com (M.F.); jiajiahu198247@163.com (J.L.); coldxiaosi@163.com (Y.J.S.); sumilhm@163.com (M.S.); gqwguoqiwei@sina.com (Q.W.G.)

**Keywords:** *TNF-RII* polymorphism, adolescents, prevalence of depression, Wenchuan earthquake

## Abstract

Objective: The aim of current study was to explore longitudinally the prevalence, severity, potential factors, and predictors of depression among Chinese Han adolescent survivors with different genotypes of *tumor necrosis factor receptor-II* (*TNF-RII*) rs1061622 after the 2008 Wenchuan earthquake. Method: *TNF-RII* rs1061622 variants were examined by polymerase chain reaction–restriction fragment length polymorphism and verified by DNA sequencing. Depression symptoms were assessed by Beck Depression Inventory (BDI) among 439 high school students at 6, 12, and 18 months after the earthquake. Results: No significant differences were observed in depression prevalence and BDI scores between the TT homozygotes and the G allele carriers in both the male and female subjects. However, the female TT homozygotes had a higher depression prevalence than the male TT homozygotes at 6, 12, and 18 months, whereas the female G allele carriers had a higher depression prevalence than the male G allele carriers only at 6 and 12 months after the earthquake. Moreover, BDI scores declined in the male subjects with both genotypes and only in the female G allele carriers at 12 months when compared with those at 6 months. Furthermore, the predictors of depression severity or potential factors of depression prevalence were different between the G allele carriers and the TT homozygotes at different times after the earthquake. Conclusion: It is concluded that the association of *TNF-RII* rs1061622 with depression is longitudinally different in Chinese Han adolescents after the 2008 Wenchuan earthquake. The T allele may be associated with reduced recovery of depression in female adolescents in the earlier stage of depression rehabilitation.

## 1. Introduction

Depression is one of the most common psychiatric disorders in adolescents all over the world [1,2], which appears after experiencing severe traumatic situations, such as earthquakes, violence, and other man-made or natural disasters. Depression affects the personal lives of the patients and results negatively in disruptions in community and society. Understanding the underlying mechanisms is essential for effective prediction and prevention of depression [3,4,5]. Previous studies indicated that natural disasters had a significant influence on the percentage of adolescents experiencing depression [6], which included earthquakes [7,8,9]. These differences in depression prevalence after earthquakes across the surveys might be due to the degree of damages, the timing of psychiatric assessments, study populations, and other factors. However, genetic backgrounds have not been included in the available literature, although genetic predisposition is very important for the prevalence of depression [10,11].

The pathogenesis of depression has not been clarified yet. Studies have shown reduced hippocampus volume in patients with major depressive disorder (MDD) [12,13], which can be caused by inflammation. Tumor necrosis factor α (TNF-α), a pleiotropic cytokine, regulates many cellular processes including inflammation. It functions through binding and activating of tumor necrosis factor receptor I (*TNF-RI*) and *TNF-RII* [14]. In fact, it has been documented that *TNF-RII* is related with depression [15]. Increased mRNA levels of TNF-α and *TNF-RII* in lymphocytes were found for depression patients [16], and increased levels of serum *TNF-RII* in heart failure and depression patients [17]. However, no significant correlation was found in the levels of TNF-α among depressed female patients [18]. Moreover, after the adjustment for anti-inflammatory drugs and other covariates, no significant association was found between *TNF-RII* and depression [19]. Due to the inconsistencies of previous results, extensive research needs to be conducted to confirm the inter-relationships between *TNF-RII* and depression.

The *TNF-RII* gene (*TNF-RII*, *CD120b* and *TNF-R P75/80*) is located on chromosome 1p36.2 and comprises 10 exons and 9 introns. Based on published sequences, biallelic polymorphisms have been detected in exons 4, 6, 9, and 10 of *TNF-RII* [20]. Among them, rs1061622 (T676G) at exon 6 is one of the most widely-studied polymorphisms. The *TNF-RII* rs1061622 mutation is the substitution from T to G at 676 nucleotide, leading to an amino acid variation in the fourth extracellular cysteine-rich domain (CRD4) from methionine to arginine at position 196 in the protein sequence [20]. Previous studies have demonstrated that *TNF-RII* rs1061622 is associated with psychological or psychiatric disorders. Hohjoh et al. [21] observed that the frequency of *TNF-RII* rs1061622 G allele was considerably higher in narcoleptic patients compared with control subjects. Moreover, the frequencies of GG genotype or G allele of *TNF-RII* rs1061622 were higher in patients with paranoid schizophrenia in comparison with healthy controls [22]. However, no studies have been conducted on the linkages between this polymorphism and depression.

An extensive literature survey identified variations in depression prevalence after earthquakes and changes in pathophysiological mechanisms of depression. We hypothesized that *TNF-RII* rs1061622 might interact with other predictors or potential factors to influence depression differently at different times in the course after stress. To test our hypothesis, depression was examined in high school students with different genotypes of *TNF-RII* rs1061622 at 6, 12, and 18 months after the 2008 Wenchuan earthquake (magnitude 8.0 on Richter scale) in China. To our best knowledge, the present work is the first attempt to explore the longitudinal relationship between *TNF-RII* rs1061622 and depression.

## 2. Subjects and Methods

### 2.1. Study Population

Study population was selected from a public boarding high school 10 km away from the epicenter and seriously affected by the earthquake in 2008. The teaching halls and student dormitories were heavily damaged. They stayed in shelters for about 15 months after the earthquake until new dormitories and teaching halls were reconstructed. Inclusion criteria for the survey were understanding the procedures involved, providing written consent from the participants and their guardians, providing blood samples, and finishing all the questionnaires during follow-ups. The exclusion criteria included: (1) students with cardiovascular, renal, and endocrinological diseases and diabetes, (2) students who took lipid-lowering drugs or hormones, consumed alcohol and smoked, (3) the students who did not finish all the questionnaires during follow-up after the earthquake, (4) the students who gave more than one answer for any single answer questions in the questionnaires, (5) no answers were chosen for any question. A total of 746 Chinese Han students of grade 11 were selected for the study as there was enough time for the follow-up before their graduation. Amongst the participants, 439 students were finally included in the study. Prior to the beginning of the survey, written consent was obtained from the participants and their guardians. Moreover, the Ethics Committee of Sichuan University gave approval for the study.

### 2.2. Measurements

The measurements were carried out at 6, 12, and 18 months after the earthquake, and were comprised of two sections [23]. The first section included the collection of demographic characters, evaluation of individual traumatic characteristics and family back ground. The demographic features were assessed through questionnaires which included gender, age, number of family members, one-child, school accommodation and education of the parents. The education of parents was classified as postgraduate, undergraduate, high school, secondary school, and illiterate. Self-reported scales were used to assess the trauma characteristics such as the number of family individuals’ deaths or injuries, the degree of property and home damages. The degree of damages to property and home was categorized as severe, moderate, minimal damage, and no loss. Personal and family history was comprised of individual and family psychiatric background and previous trauma experience.

The second section included the Beck Depression Inventory (BDI), for the examination of depression severity [24]. Previously published guidelines for the application of the instrument were followed accordingly [25]. The Chinese version of the BDI has been applied due to its reliability, convergent validity and accurate results [26]. It is comprised of 21 questions and the score of each question is between 0 and 3. The total scores range from 0 to 63 [27]. The cut-off score of 14 was used to categorize whether subjects had depressive symptoms or not [27]. The value of Cronbach’s α coefficient ranged from 0.811 to 0.912 in the current study [23].

### 2.3. Extraction of DNA and Genotype Analysis

Genomic DNA was isolated from blood peripheral leucocytes through a commercial DNA extraction kit (Tiandz, china) at 18 months after the earthquake and stored at −80 °C for further uses. The polymorphism of *TNF-RII* rs1061622 was determined by polymerase chain reaction–restriction fragment length polymorphism (PCR-RFLP) in 2017. Results of genotyping were confirmed by DNA sequencing. A 242 base pair fragment including *TNF-RII* rs1061622 was amplified by the following primers [28]: forward, 5′-ACT CTC CTA TCC TGC CTG CT-3′; and reverse, 5′-TTC TGG AGT TGG CTGCGT GT-3′. An amount of 2 µL of PCR products were consequently digested with 1 µL *Nla* III restriction enzymes overnight. Digested products were separated through 3% agrose gel electrophoresis. Wild type homozygotes (TT) were shown as two bands (133bp and 109bp), heterozygotes (GT) as three bands (242 bp, 133 bp and 109 bp), and mutant homozygotes (GG) as a single band (242 bp) [29].

### 2.4. Statistical Analysis

We used chi-squared goodness-of-fit to assess the genotype distribution with Hardy-Weinberg equilibrium. Chi-square tests were used to analyze genotype distributions and depression prevalence between genders, the subjects at different time durations, and the subjects with different genotypes of *TNF-RII* rs1061622. BDI scores of the subjects with different genders, the subjects at different times after the earthquake, or the subjects with different genotypes of *TNF-RII* rs1061622 were analyzed using Friedman M and Kruskal Wallis tests. Independent predictors of depression severity were identified using stepwise multiple linear regression analysis. Impacts of independent variables on depression prevalence were assessed using binary logistic analysis. *p* ≤ 0.05 was defined as statistically significant.

## 3. Results

### 3.1. Frequencies of the Genotypes and Alleles of TNF-RII rs1061622

Table 1 presents the genotypic and allelic frequencies of *TNF-RII* rs1061622 in the population. Genotypic distribution of *TNF-RII* rs1061622 did not show any deviation (χ^2^ = 0.686, *p* = 0.710) according to Hardy-Weinberg Equilibrium. Genotypic frequencies of *TNF-RII* rs1061622 were not significantly different between both genders in the study population (*p* = 0.704).

### 3.2. Prevalence of Depression for the Subjects with Different Genotypes of TNF-RII rs1061622 at 6, 12, and 18 Months after the Earthquake

Due to the insufficient number, the GG homozygotes of *TNF-RII* rs1061622 were taken together with the GT heterozygotes and were labeled as the G allele carriers for further analyses. Prevalence of depression for the subjects with different genotypes of *TNF-RII* rs1061622 is shown in Table 2. There were no notable differences of depression prevalence between the TT homozygotes and the G allele carriers at 6, 12, and 18 months in the students, irrespective of gender. There were no significant changes in depression prevalence during the follow-up in both genders regardless of the genotypes. However, the depression prevalence decreased significantly at 18 months compared with 6 months in the TT homozygotes in the whole study population (*p* = 0.010). When analyzed between different genders, the females had a higher prevalence than the males, irrespective of the genotypes at 6 (TT homozygotes: *p* = 0.001; G allele carries: *p* = 0.033) and at 12 months (TT homozygotes: *p* = 0.001; G allele carries: *p* = 0.049). However, a higher prevalence was found in the females than the males in the TT homozygotes (*p* = 0.014), but not in the G allele carries at 18 months after the earthquake.

### 3.3. BDI Scores of the Subjects with Different Genotypes of TNF-RII rs1061622 at 6, 12, and 18 Months after the Earthquake

BDI scores of the subjects with different genotypes are shown in Table 3. There were no significant differences between the TT homozygotes and the G allele carriers at 6, 12, and 18 months in the whole study population. However, in the whole study population the BDI scores decreased significantly at 12 (TT homozygotes: *p* = 0.004; G allele carriers: *p* = 0.001) and at 18 months (TT homozygotes: *p* < 0.001; G allele carriers: *p* < 0.001) when compared with 6 months. The scores also declined at 18 months (TT homozygotes: *p* < 0.001; G allele carriers: *p* = 0.002) when compared with 12 months after the earthquake.

When gender was taken into consideration, BDI scores were significantly lower at 12 months when compared with 6 months in the males with both genotypes (TT homozygotes: *p* = 0.020; G allele carriers: *p* = 0.004), and in the female G allele carriers (*p* = 0.047), but not in female TT homozygotes. In contrast, scores decreased at 18 months when compared with 12 months (TT homozygotes: *p* = 0.018; G allele carriers: *p* = 0.011) and 6 months (TT homozygotes: *p* < 0.001; G allele carriers: *p* < 0.001) in the male students irrespective of the genotypes. In female students, BDI scores significantly decreased at 18 months in both genotypes when compared with 6 months (TT homozygotes: *p* < 0.001; G allele carriers: *p* = 0.001) and 12 months (TT homozygotes: *p* < 0.001; G allele carriers: *p* = 0.045) after the earthquake.

In addition, the female subjects had higher BDI scores than the males at 6 months (TT homozygotes: *p* < 0.001; G allele carriers: *p* = 0.032), at 12 months (TT homozygotes: *p* < 0.001; G allele carriers: *p* = 0.018), and at 18 months (TT homozygotes: *p* < 0.001; G allele carriers: *p* = 0.010) after the earthquake regardless of the genotypes.

### 3.4. Potential Factors Associated with Depression Prevalence in the Subjects with Different Genotypes of TNF-RII rs1061622 at 6, 12, and 18 Months after the Earthquake

The predictive factors of depression prevalence in different genotypes of *TNF-RII* rs1061622 are shown in Table 4. In the TT homozygotes, gender (*p* < 0.001), age (*p* = 0.012), and direct exposure to a family member’s death, injury or home damage (*p* = 0.038) were the predictive factors of depression prevalence at 6 months. At 12 months, gender (*p* < 0.001), age (*p* = 0.041) and previous trauma experience (*p* = 0.040) were the predictive factors of depression prevalence. Gender (*p* = 0.005) and age (*p* = 0.019) were also the predictors at 18 months.

In the G allele carriers, gender (6 months: *p* = 0.006; 12 months: *p* = 0.010) and self-injury (6 months: *p* = 0.030; 12 months: *p* = 0.030), were the predictors of depression prevalence at 6 and 12 months, respectively. At 18 months, gender (*p* = 0.049) and age (*p* = 0.033) were the significant potential factors of depression prevalence.

### 3.5. Predictors of BDI Scores in the Subjects with Different Genotypes of TNF-RII rs1061622 at 6, 12, and 18 Months after the Earthquake

Stepwise multivariate linear regression analyses for predictors of depression severity in the subjects with different genotypes of *TNF-RII* rs1061622 at 6, 12, and 18 months are shown in Table 5. In the TT homozygotes, gender, previous trauma experience, extent of home damage, direct exposure to a family member’s death or injury or home damage, and age accounted for 8.2%, 2.9%, 2.1%, 2.0%, and 1.6%, respectively, of the variance of depression severity at 6 months. At 12 and 18 months, gender, age, and previous trauma experience were also the predictors of depression severity, which accounted for 7.9%, 3.9%, and 2.2% (12 months), and 4.7%, 2.9%, and 1.9% (18 months) of the variance, respectively.

In the G allele carriers, gender; self-injury; direct exposure to a family member’s injury, death, or home damage; and family individual injury were predictors of depression severity, which accounted for 7.2%, 5.6%, 3.0%, and 2.9% of variance, respectively, at 6 months. At 12 and 18 months, self-injury and gender were also predictors of depression severity and accounted for 10.1% and 8.0% (12 months), and 6.0% and 5.9% (18 months) of variance, respectively.

## 4. Discussion

The developing mechanism of depression has not been completely understood yet. Pro-inflammatory cytokines were found in both peripheral and central regions and acted to trigger sickness behaviors [30,31], including decreased social behavior, feeding, and locomotor activity, which were linked to symptoms of major depressive disorder [30]. Therefore, it is critical to note the relationship of pro-inflammatory cytokines and their receptors with depression. TNF-RII is the receptor of TNF-α, a pro-inflammatory cytokine [32]. Simen et al. [33] reported that TNF-α could induce depression-like symptoms even in the absence of malaise and demonstrated that TNF-RII might be involved in this response. Previous studies also demonstrated that higher levels of plasma TNF-RII were found in the female patients with MDD compared with healthy controls. Similarly, TNF-RII levels were also higher in bipolar depression individuals as compared with the healthy subjects [18,34]. Furthermore, higher mRNA levels of TNF-α and TNF-RII were found in the lymphocytes of depressive patients when compared with control subjects [16]. A meta-analysis showed that depressive patients had higher levels of TNF-α compared with controls [35]. These studies suggest that depression may be at least partly related to TNF-RII.

Although the logistic regression model confirmed the increased risk of paranoid schizophrenia with the inheritance of the G allele of *TNF-RII* rs1061622 [22], the association of this polymorphism with depression has not been explored yet. Therefore, the present work was performed, for the first time to the best of our knowledge, to analyze the prevalence and severity of depression, and their potential factors and predictors in the subjects with different genotypes of *TNF-RII* rs1061622 among adolescent survivors longitudinally at 6, 12, and 18 months after the Wenchuan earthquake. The present results showed non-significant differences in depression prevalence (Table 2) and BDI scores (Table 3) between the TT homozygotes and the G allele carriers in the male or female students at 6, 12, and 18 months after the earthquake. The female subjects had higher BDI scores than the males at 6, 12, and 18 months after the earthquake regardless of the genotypes (Table 3). However, the female students had a higher prevalence of depression than the male students only in the TT homozygotes, but not in the G allele carries at 18 months (Table 2). BDI scores were observed to be decreased in the male students with both genotypes and the female G allele carriers at 12 months in comparison with those at 6 months (Table 3). In addition, the potential factors of depression prevalence or predictors of depression severity were different between G allele carriers and TT homozygotes at different times after the earthquake (Table 4 and Table 5). Gender, previous trauma experience, degree of home damage, direct exposure to a family member’s death, injury or home damage, and age were the predictors of BDI scores in the TT homozygotes at 6 months. The predictors of depression severity at 6 months in the G allele carriers were gender, self-injury, direct exposure to a family member’s death, injury or home damage, and family individual injury. Other differences of potential factors or predictors were also observed between the TT homozygotes and the G allele carriers at 12 and 18 months after the earthquake (Table 4 and Table 5). These results suggest that there may be different interplays among gender, *TNF-RII* rs1061622, and other predictors or potential factors at different times after the stress induced by the earthquake to affect depression, although the effects may be minor.

The *TNF-RII* rs1061622 has showed no effect on TNF binding kinetics with TNF-RII. However, the increased frequency of G allele of *TNF-RII* rs1061622 transduced the signals of TNF-α more effectively than the T allele, without affecting the binding affinity of TNF-α to TNF-RII [36]. The mutation of *TNF-RII* rs1061622 resulted in a significantly lower capability to induce TNF-RII-mediated NF-κB activation and the expression of NF-κB dependent target genes conveying anti-apoptotic or pro-inflammatory functions [37]. Interface amongst TNFR-II and glutamate induces extended activation of phosphatidylinositol-3-kinase–dependent NF-κB activation that improves neuronal survival and modulates sensitivity to excitotoxic stress, including that which happens in TBI (traumatic brain injury) and contributes to motor and cognitive dysfunction [38]. The polymorphism of TNFR-II was also found to be associated with the susceptibility to inflammatory diseases, and psychological disorders [21,22,39]. Therefore, the effects of interplays observed in the present study may be associated with the capability changes of mutant TNF-RII to induce TNF-RII-mediated NF-κB activation and the expression of NF-κB dependent target genes. This association needs to be confirmed in future studies.

These results in the present study demonstrate that *TNF-RII* rs1061622 may interact with other predictors and potential factors to affect the prevalence and severity of depression differently at different time durations after the earthquake. This can be one of the explanations for the drastic variations in depression prevalence after earthquakes.

## 5. Conclusions

Significant decreases in BDI scores were observed in both genotypes of males and only the G allele carriers of females at 12 months when compared with those at 6 months after the earthquake. This result suggests delayed rehabilitation from depression in Chinese female adolescents carrying the T allele of *TNF-RII* rs1061622 in the earlier stage of the follow-up after the Wenchuan earthquake. Furthermore, different patterns of predictors or potential factors were demonstrated between the G allele carriers and the TT homozygotes of *TNF-RII* rs1061622 at different times after the earthquake. *TNF-RII* rs1061622 may interplay with other predictors and potential factors to affect depression after stress induced by the earthquake in a gender- and time-dependent manner. These results provide novel insights into the mechanism of depression in terms of TNF-RII and its interplays with other predictors and potential factors.

## 6. Limitations

The present study had some limitations. First, the levels of TNF-RII in serum were not measured. Second, the effects of body mass index, sex hormones, and menstruation were not tested. Third, the exclusion of students who had consumed alcohol or smoked might have impacts on the results since there might be a link between these addictions and depression.

## Figures and Tables

**Table 1:** Allele and genotype frequencies of *tumor necrosis factor receptor-II* (*TNF-RII*) rs1061622 in the study population.

	Total (*n* = 439)	Males (*n* = 197)	Females (*n* = 242)
	*n* (%)	*n* (%)	*n* (%)
Genotype frequencies			
TT	294 (66.97)	135 (68.53)	159 (65.70)
GT	126 (28.70)	55 (27.92)	71 (29.34)
GG	19 (4.33)	7 (3.55)	12 (4.96)
Allele frequencies			
T	714 (81.32)	325 (82.49)	389 (80.37)
G	164 (18.68)	69 (17.51)	95 (19.63)

**Table 2:** Prevalence of depression for the subjects with different genotypes of *TNFRII* rs1061622 at 6, 12, and 18 months after the earthquake.

Time	Males		Females		All	
	TT	G Allele Carriers	TT	G Allele Carriers	TT	G Allele Carriers
6 months	36 (26.67)	19 (30.65)	72 (45.28) ^###^	40 (48.19) ^#^	108(36.73)	59 (40.69)
12 months	32(23.70)	15 (24.19)	68 (42.77) ^###^	33 (39.76) ^#^	100 (34.01)	48 (33.10)
18 months	27(20.00)	13 (20.97)	52 (32.70) ^#^	28 (33.73)	79 (26.87) **	41 (28.28)

Data are expressed as *n* (%), ** *p* < 0.01 when compared with that of the TT homozygotes at 6 months after the earthquake (Chi-square tests), ^#^ *p* ≤ 0.05, and ^###^ *p* < 0.001 when compared with that of the males in the same genotype at the same time after the earthquake (Chi-square tests).

**Table 3:** Beck Depression Inventory (BDI) scores of the subjects with different genotypes of *TNFRII* rs1061622 at 6, 12, and 18 months after the earthquake.

Time	Males		Females		All	
	TT	G Allele Carriers	TT	G Allele Carriers	TT	G allele Carriers
6 months	9.00 (4.00–14.00)	10.00 (5.00–16.00)	13.00 (8.00–19.00) ^&&&^	13.00 (7.00–20.00) ^&^	11.00 (6.00–16.00)	12.00 (6.00–18.00)
12 months	7.00 (2.00–13.00) *	9.00 (4.00–13.25) **	11.00 (6.00–18.00) ^&&&^	11.00 (6.00–19.00) *^,&^	9.00 (5.00–17.00) **	10.00 (4.00–16.00) ***
18 months	5.00 (1.00–11.00) ***^,#^	5.50 (2.75–11.25) ***^,#^	9.00 (4.00–17.00) ***^,###,&&&^	10.00 (4.00–17.00) ***^,#,&&^	7.50 (3.00–14.75) ***^,###^	8.00 (4.00–16.00) ***^,##^

Data are expressed as median (inter quartile range). * p ≤ 0.05, ** *p* < 0.01, and *** *p* < 0.001 when compared with those of the subjects with the same genotype at 6 months after the earthquake (Friedman M test), ^#^ *p* ≤ 0.05, ^##^ *p* < 0.01, and ^###^ *p* < 0.001 when compared with those of the subjects with the same genotype at 12 months after the earthquake (Friedman M test), ^&^ *p* ≤ 0.05, ^&&^ *p* < 0.01, and ^&&&^ *p* < 0.001 when compared with those of the male subjects with the same genotype at the same months after the earthquake (Kruskal Wallis test).

**Table 4:** Potential factors associated with depression prevalence in the subjects with different genotypes of *TNFRII rs1061622* at 6, 12, and 18 months after the earthquake.

Variables	6 Months				12 Months				18 Months			
	TT		GG Allele Carriers		TT		GG Allele Carriers		TT		GG Allele Carriers	
	Adjusted OR	95% CI	Adjusted OR	95% CI	Adjusted OR	95% CI	Adjusted OR	95% CI	Adjusted OR	95% CI	Adjusted OR	95% CI
Gender ^a^	3.31 ***	1.85–5.94	3.15 **	1.40–7.07	3.14 ***	1.75–5.66	3.14 **	1.31–7.53	2.43 **	1.31–4.51	2.39 *	1.00–5.69
Age ^b^	1.91 *	1.15–3.19	--	--	1.69 *	1.02–2.79	--	--	1.91 *	1.11–3.28	0.39 *	0.16–0.93
Direct exposures ^c^	0.55 *	0.31–0.97	--	--	--	--	--	--	--	--	--	--
Self-injury ^c^	--	--	11.49 *	1.27–104.3	--	--	7.04 *	1.21–40.83	--	--	--	--
Previous trauma experience ^c^	--	--	--	--	8.74 *	1.11–69.04	--	--	--	--	--	--
Constant ^b^	0.00 **		0.30 ***		0.00 **		0.20 ***		0.00 **		0.02 *	

Note. OR, odds ratio; CI, confidence interval; Direct exposures, direct exposures to family member’s injury or death or home damage; --, no significant result was found; ^a^, 0 = male, 1 = female; ^b^, included in model as continuous variable; ^c^, 0 = no, 1 = yes; * p ≤ 0.05, ** p < 0.01, and *** p < 0.001.

**Table 5:** Predictors of BDI scores in the subjects with different genotypes of *TNFRII rs1061622* at 6, 12, and 18 months after the earthquake.

Variables	6 Months				12 Months				18 Months			
	TT		G Allele Carriers		TT		G Allele Carriers		TT		G Allele Carriers	
	Adjusted R^2^ = 0.15		Adjusted R^2^ = 0.16		Adjusted R^2^ = 0.13		Adjusted R^2^ = 0.17		Adjusted R^2^ = 0.08		Adjusted R^2^ = 0.10	
	β ^a^	Partial Correlations	β ^a^	Partial Correlations	β ^a^	Partial Correlations	β ^a^	Partial Correlations	β ^a^	Partial Correlations	β ^a^	Partial Correlations
Gender	0.26 ***	0.27 ***	0.25 **	0.27 **	0.29 ***	0.30 ***	0.28 ***	0.30 ***	0.22 ***	0.23 ***	0.24 **	0.25 **
Age	0.13 *	0.14 *	--	--	0.20 ***	0.21 ***	--	--	0.17 **	0.18 **	--	--
Previous trauma experience	0.15 *	0.16 *	--	--	0.15 *	0.16 *	--	--	0.14 *	0.14 *	--	--
Home damage	0.16 *	0.17 *	--	--	--	--	--	--	--	--	--	--
Direct exposures	−0.13 *	−0.14 *	0.20 *	0.21 *	--	--	--	--	--	--	--	--
Self-injury	--	--	0.20 *	0.21 *	--	--	0.32 ***	0.33 ***	--	--	0.24 **	0.25 **
Family individual injury	--	--	0.18 *	0.19 *	--	--	--	--	--	--	--	--

**β**^a^ Standardized regression coefficient; Direct exposures, direct exposures to family member’s injury or death or home damage; --, no significant result was found. * *p* ≤ 0.05, ** *p* < 0.01, and *** *p* < 0.001.

## Abbreviations

BDI	Beck depression inventory;
CRD4	Fourth extracellular cysteine-rich domain;
MDD	Major depressive disorder;
PCR-RFLP	Polymerase chain reaction–restriction fragment length polymorphism;
TNF-α	Tumor necrosis factor-α;
*TNF-RI*	Tumor necrosis factor-α receptor;
*TNF-RII*	Tumor necrosis factor receptor-II.

## References

[1] Primo de Carvalho Alves L., Pio de Almeida Fleck M., Boni A., Sica da Rocha N. (2017). The Major Depressive Disorder Hierarchy: Rasch Analysis of 6 items of the Hamilton Depression Scale Covering the Continuum of Depressive Syndrome. PLoS ONE.

[2] Wesselhoeft R., Sørensen M.J., Heiervang E.R., Bilenberg N. (2013). Subthreshold depression in children and adolescents—A systematic review. J. Affect. Disord..

[3] Lê F., Tracy M., Norris F.H., Galea S. (2013). Displacement, county social cohesion, and depression after a large-scale traumatic event. Soc. Psychiatry Psychiatr. Epidemiol..

[4] Karam E.G., Fayyad J., Karam A.N., Melhem N., Mneimneh Z., Dimassi H., Tabet C.C. (2014). Outcome of depression and anxiety after war: A prospective epidemiologic study of children and adolescents. J. Trauma. Stress.

[5] Lai B.S., La Greca A.M., Auslander B.A., Short M.B. (2013). Children’s symptoms of posttraumatic stress and depression after a natural disaster: Comorbidity and risk factors. J. Affect. Disord..

[6] Tang B., Liu X., Liu Y., Xue C., Zhang L. (2014). A meta-analysis of risk factors for depression in adults and children after natural disasters. BMC Public Health.

[7] Kadak M.T., Nasıroğlu S., Boysan M., Aydın A. (2013). Risk factors predicting posttraumatic stress reactions in adolescents after 2011 Van earthquake. Compr. Psychiatry.

[8] Chui C.H.K., Ran M.-S., Li R.-H., Fan M., Zhang Z., Li Y.-H., Ou G.J., Jiang Z., Tong Y.-Z., Fang D.-Z. (2017). Predictive factors of depression symptoms among adolescents in the 18-month follow-up after Wenchuan earthquake in China. J. Ment. Health.

[9] Shi X., Yu N.X., Zhou Y., Geng F., Fan F. (2016). Depressive symptoms and associated psychosocial factors among adolescent survivors 30 months after 2008 Wenchuan earthquake: A follow-up study. Front. Psychol..

[10] Wang Q., Zhu X.C., Liu H., Ran M.S., Fang D.Z. (2015). A longitudinal study of the association of adiponectin gene rs1501299 with depression in Chinese Han adolescents after Wenchuan earthquake. J. Affect. Disord..

[11] McGuffin P., Katz R., Watkins S., Rutherford J. (1996). A hospital-based twin register of the heritability of DSM-IV unipolar depression. Arch. Gen. Psychiatry.

[12] Gerritsen L., van Velzen L., Schmaal L., van der Graaf Y., van der Wee N., van Tol M.J., Penninx B., Geerlings M. (2015). Childhood maltreatment modifies the relationship of depression with hippocampal volume. Psychol. Med..

[13] Abdallah C.G., Jackowski A., Sato J.R., Mao X., Kang G., Cheema R., Coplan J.D., Mathew S.J., Shungu D.C. (2015). Prefrontal cortical GABA abnormalities are associated with reduced hippocampal volume in major depressive disorder. Eur. Neuropsychopharmacol..

[14] Wajant H., Pfizenmaier K., Scheurich P. (2003). Tumor necrosis factor signaling. Cell Death Differ..

[15] O’Donovan A., Chao L.L., Paulson J., Samuelson K.W., Shigenaga J.K., Grunfeld C., Weiner M.W., Neylan T.C. (2015). Altered inflammatory activity associated with reduced hippocampal volume and more severe posttraumatic stress symptoms in Gulf War veterans. Psychoneuroendocrinology.

[16] Rizavi H.S., Ren X., Zhang H., Bhaumik R., Pandey G.N. (2016). Abnormal gene expression of proinflammatory cytokines and their membrane-bound receptors in the lymphocytes of depressed patients. Psychiatry Res..

[17] Moughrabi S., Evangelista L.S., Habib S.I., Kassabian L., Breen E.C., Nyamathi A., Irwin M. (2014). In patients with stable heart failure, soluble TNF-receptor 2 is associated with increased risk for depressive symptoms. Biol. Res. Nurs..

[18] Grassi-Oliveira R., Brietzke E., Pezzi J.C., Lopes R.P., Teixeira A.L., Bauer M.E. (2009). Increased soluble tumor necrosis factor-α receptors in patients with major depressive disorder. Psychiatry Clin. Neurosci..

[19] Chocano-Bedoya P.O., Mirzaei F., O’Reilly E.J., Lucas M., Okereke O.I., Hu F.B., Rimm E.B., Ascherio A. (2014). C-reactive protein, interleukin-6, soluble tumor necrosis factor α receptor 2 and incident clinical depression. J. Affect. Disord..

[20] Pantelidis P., Lympany P.A., Foley P.J., Fanning G.C., Welsh K.I., du Bois R.M. (1999). Polymorphic analysis of the high-affinity tumor necrosis factor receptor 2. Tissue Antigens.

[21] Hohjoh H., Terada N., Kawashima M., Honda Y., Tokunaga K. (2000). Significant association of the tumor necrosis factor receptor 2 (TNFR2) gene with human narcolepsy. Tissue Antigens.

[22] Thabet S., Ben Nejma M., Zaafrane F., Gaha L., Ben Salem K., Romdhane A., Nour M., Bel Hadj Jrad B. (2011). Association of the Met-196-Arg variation of human tumor necrosis factor receptor 2 (TNFR2) with paranoid schizophrenia. J. Mol. Neurosci..

[23] Zhang Z., Ran M.S., Li Y.H., Ou G.J., Gong R.R., Li R.H., Fan M., Jiang Z., Fang D.Z. (2012). Prevalence of post-traumatic stress disorder among adolescents after the Wenchuan earthquake in China. Psychol. Med..

[24] Beck A.T., Ward C.H., Mendelson M.M., Mock J.J., Erbaugh J.J. (1961). An inventory for measuring depression. Arch. Gen. Psychiatry.

[25] Brislin R.W. (1976). Translation: Applications and Research.

[26] Shek D.T.L. (1991). What does the Chinese version of the Beck Depression Inventory measure in Chinese students—General psychopathology or depression?. J. Clin. Psychol..

[27] Wang Y., Chan R.C.K., Deng Y. (2006). Examination of postconcussion-like symptoms in healthy university students: Relationships to subjective and objective neuropsychological function performance. Arch. Clin. Neuropsychol..

[28] Glossop J.R., Dawes P.T., Nixon N.B., Mattey D.L. (2005). Polymorphism in the tumour necrosis factor receptor II gene is associated with circulating levels of soluble tumour necrosis factor receptors in rheumatoid arthritis. Arthritis Res. Ther..

[29] Said L., Faleh R., Smida S., Laajili H., Sakouhi M., Bel Hadj Jrad B. (2013). Maternal tumor necrosis factor receptor 2 gene variants associated with pre-eclampsia in Tunisian women. J. Obstet. Gynaecol. Res..

[30] Dantzer R. (2004). Innate immunity at the forefront of psychoneuroimmunology. Brain Behav. Immun..

[31] Bluthé R.-M., Layé S., Michaud B., Combe C., Dantzer R., Parnet P. (2000). Role of interleukin-1β and tumour necrosis factor-α in lipopolysaccharide-induced sickness behaviour: A study with interleukin-1 type I receptor-deficient mice. Eur. J. Neurosci..

[32] Chu W.-M. (2013). Tumor necrosis factor. Cancer Lett..

[33] Simen B.B., Duman C.H., Simen A.A., Duman R.S. (2006). TNFalpha signaling in depression and anxiety: Behavioral consequences of individual receptor targeting. Biol. Psychiatry.

[34] Teixeira A.L., de Souza R.T., Zanetti M.V., Brunoni A.R., Busatto G.F., Zarate C.A., Gattaz W.F., Machado-Vieira R. (2015). Increased plasma levels of soluble TNF receptors 1 and 2 in bipolar depression and impact of lithium treatment. Hum. Psychopharmacol. Clin..

[35] Dowlati Y., Herrmann N., Swardfager W., Liu H., Sham L., Reim E.K., Lanctôt K.L. (2010). A meta-analysis of cytokines in major depression. Biol. Psychiatry.

[36] Morita C., Horiuchi T., Tsukamoto H., Hatta N., Kikuchi Y., Arinobu Y., Otsuka T., Sawabe T., Harashima S., Nagasawa K. (2001). Association of tumor necrosis factor receptor type II polymorphism 196R with systemic lupus erythematosus in the Japanese: Molecular and functional analysis. Arthritis Rheum..

[37] Till A., Rosenstiel P.C., Krippner-Heidenreich A., Mascheretti-Croucher S., Croucher P.J.P., Schäfer H., Scheurich P., Seegert D., Schreiber S. (2005). The Met196Arg variation of human TNFR2 affects TNF-α-induced apoptosis by impaired NF-κB-signalling and target gene expression. J. Biol. Chem..

[38] Marchetti L., Klein M., Schlett K., Pfizenmaier K., Eisel U.L. (2004). Tumor necrosis factor (TNF)-mediated neuroprotection against glutamate-induced excitotoxicity is enhanced by N-methyl-D-aspartate receptor activation. Essential role of a TNF receptor 2-mediated phosphatidylinositol 3-kinase-dependent NF-kappa B pathway. J. Biol. Chem..

[39] Ferguson L.R., Han D.Y., Huebner C., Petermann I., Barclay M.L., Gearry R.B., McCulloch A., Demmers P.S. (2014). Tumor necrosis factor receptor superfamily, member 1B haplotypes increase or decrease the risk of inflammatory bowel diseases in a New Zealand caucasian population. Gastroenterol. Res. Pract..

